# A compact underactuated gripper with two fingers and a retractable suction cup

**DOI:** 10.3389/frobt.2023.1066516

**Published:** 2023-04-17

**Authors:** Julien Courchesne, Philippe Cardou, Palamanga Abdoul Rachide Onadja

**Affiliations:** Laboratoire de robotique, Département de génie mécanique, Université Laval, Quebec City, QC, Canada

**Keywords:** gripper, vacuum gripper, suction cup, hybrid grasping, robot hand, underactuated, planetary gear set

## Abstract

Modern industrial applications of robotics such as small-series production and automated warehousing require versatile grippers, i.e., grippers that can pick up the widest possible variety of objects. These objects must often be grasped or placed inside a container, which limits the size of the gripper. In this article, we propose to combine the two most popular gripper technologies in order to maximise versatility: finger grippers and suction-cup (vacuum) grippers. Many researchers and a few companies have followed this same idea in the past, but their gripper designs are often overly complex or too bulky to pick up objects inside containers. Here, we develop a gripper where the suction cup is lodged inside the palm of a two-finger robotic hand. The suction cup is mounted on a retractile rod that can extend to pick up objects inside containers without interference from the two fingers. A single actuator drives both the finger and sliding-rod motions so as to minimise the gripper complexity. The opening and closing sequence of the gripper is achieved by using a planetary gear train as transmission between the actuator, the fingers and the suction cup sliding mechanism. Special attention is paid to minimise the overall gripper size; its diameter being kept to 75 mm, which is that of the end link of the common UR5 robot. A prototype of the gripper is built and its versatility is demonstrated in a short accompanying video.

## 1 Introduction

Versatile robotic grippers are needed in many a modern industrial application. In some instances, a more versatile gripper can replace two or more specialised gripping tools. This, in turn, can reduce the number of robotic arms needed to carry them. Versatile grippers are particularly useful for pick-and-place operations in just-in-time production, where a robotic arm must reliably grasp ever changing series of parts. In most small to medium size businesses, the costs and delays of designing or modifying a gripper to grasp each new part on the production line is prohibitive. So is the cost of an overly complex gripper that cannot resist continuous operation in a dusty environment. Aside from industrial pick-and-place, we should cite the case where there is no space for the robot to access multiple grippers. This may occur in assistive robotics and in space or ocean exploration, for instance. In such applications, the best gripper is generally the more versatile one, i.e., that which can grasp the largest number of objects within the targeted set. Reliability is also an important factor, as gripper failure can lead to the failure of a whole exploration, assembly or placement mission. Let us review below how reliability is obtained by minimising the number of actuators and how versatility is achieved by combining fingers with suction cups.

### 1.1 Underactuation for simplicity

Robotic hands with more than one actuator are a rarity in industry. Some authors have attributed this to the industry needs, which would mainly be limited to low-cost, robust, simple and reliable hands (Negrello et al., 2020). This does not mean that the industry needs could not be expanded if one were to invent more dexterous hands with advanced manipulation skills. Such hands could well find application in typical industrial tasks such as assembly or disassembly.

But so far, it seems that the surest way to favour the adoption of new robotic hands in industry is to avoid overly complex designs. This may explain why approximately 60% of new hand designs published from 2016 to 2018 have resorted to underactuated transmissions according to Piazza et al. (2019). With underactuation, the hand is driven by less actuators than its number of degrees of freedom, generally reducing cost and complexity. Among the underactuated hands that have been produced, we cite those devised by Laliberté and Gosselin. (2000) and Birglen. (2015), which were both commercialised by Robotiq Inc., and those proposed by (Dollar and Howe, 2010); (Townsend, 2000) and Ciocarlie et al. (2014). In these designs, the actuator torques are distributed among the phalanxes of all fingers through linkages in the case of (Laliberté and Gosselin, 2000); (Birglen, 2015), and through tendons in the cases of Dollar and Howe. (2010) and Ciocarlie et al. (2014).

In this article, we resort to the same principle but to a different end. We distribute the actuator torque not among the finger phalanxes, but between the fingers and the suction cup sliding mechanism. This allows us to drive these two gripping modes with a single motor.

### 1.2 Technology combination for versatility

Using multiple gripping modes allows for more versatility. At the moment, the two most effective gripper technologies in industry are certainly finger grippers and vacuum grippers. Finger grippers are those that offer the most robust grasps, which in turn allow for large accelerations of the payload once it is secured. They generally require access to the sides of the grasped object, however, which can be a problem when it is to be closely stacked with other objects or is inside a container. Finger grippers also have difficulty seizing thin objects lying flat on a horizontal surface. Vacuum grippers resolve these issues by requiring access to only one smooth surface of the object. On the other hand, their grasps are generally weaker than those of finger grippers, often limiting their payload and the accelerations they can sustain.

From these observations, it seems that the most versatile gripper could be designed by combining finger grippers with vacuum grippers. In doing so, one should take care to retain the functionalities of both technologies while avoiding their drawbacks and also limiting the added complexity. Such a gripper would be able to perform vacuum grasps when working in cluttered environments and finger grasps when high payloads or accelerations are needed. Ideally, one would be able to switch between vacuum and finger grasps on the fly or even perform *combined grasps* for increased robustness.

**FIGURE 1:**
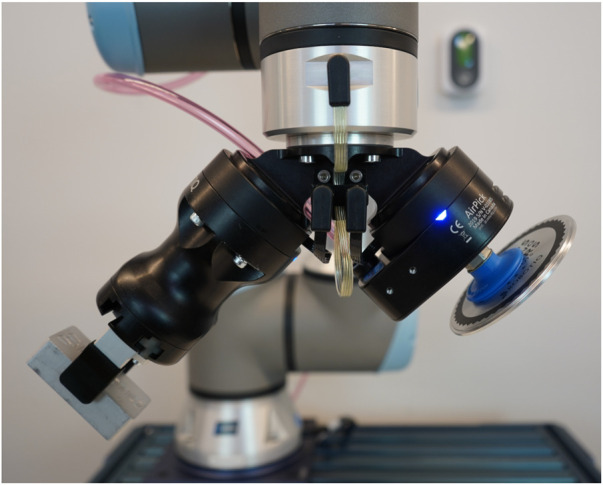
Image source: Robotiq Inc., 2022. Used with permission. The *Dual-Grip* system with the Hand-E and the AirPick grippers.

Several solutions combining a finger gripper with suction cups have been proposed in the past. Surely the most obvious of those consists in having two grippers mounted separately on the same end effector. This simple design, shown in Figure 1, was commercialised by Robotiq. (2020a) as the *Dual-Grip*. The end effector takes the shape of a “Y”, and the finger gripper and vacuum gripper are respectively mounted on each of the two top branches of the “Y”. This crude design minimises complexity in that it allows to combine off-the-shelf grippers, but it does not allow for grasps combining the two technologies and its bulkiness prevents its application to tight spaces.

Moving away from the dual-gripper solution, Bryan et al. (2019) integrated suction cups and fingers in one single gripper. In their design, the gripper is made of three wire-driven compliant fingers, each comprising three phalanxes. There is one suction cup in each phalanx to increase the adhesion of the fingers onto the grasped objects and improve the grasp robustness in general. While this hand design probably leads to good versatility and adaptability to many object shapes, it may not be the best suited for industrial applications. In such applications, accuracy and durability are important, and these qualities are difficult to achieve with soft fingers. Furthermore, a hand with nine suction cups appears overly complex when compared with the industrial robotic grippers currently on the market.

Hughes and Deis. (2020) and Zeng et al. (2018) proposed to append a single suction cup at the side of a finger gripper. This makes for a simpler solution than the soft gripper by Bryan et al. (2019), and it is more integrated than the dual gripper commercialised by Robotiq. (2020a). Still, it separates the two grasping methods as it does not allow for combined grasps. In the case of Zeng et al. (2018), the suction cup is mounted on a slider parallel to the gripper axis. This allows it to reach beyond the finger tips, and gives it access to the corners of a container. We call this design a parallel gripper, as its two distal phalanxes remain parallel throughout its closing and opening sequences. While the hand proposed by Zeng et al. (2018) can grasp a wide variety of objects, there is still room for improvement in its design. Firstly, the gripper appears to be rather long, although no dimension is given in Zeng et al. (2018). A longer gripper entails less dexterity at its fingertips and larger moments on its supporting robotic arm. Secondly, the weight may be increased by having two separate actuators for the motions of the fingers and the suction cup, respectively, although this is not a clear advantage. Thirdly and most importantly, with the proposed configuration, combined grasps appear to be impossible, i.e., one must choose between using the fingers and the suction cup for each new object. Some situations would benefit from combined grasps, however, such as those that require picking a flat object and then securing it in a robust grasp. It would thus appear desirable to relocate the suction cup in between the two fingers, a simple change in design that would allow for combined grasps.

This possibility was explored in references (Chiu et al., 2013; Hasegawa et al., 2017), and Kang et al. (2019), which present three gripper designs with a suction cup mounted in the palm, in between the fingers. Chiu et al. (2013) proposed a four-finger hand with a extensible suction cup to harvest tomatoes in a greenhouse. Each finger is driven independently by a tendon and a solenoid, while the suction cup extension is driven by a pneumatic cylinder. The design appears to be well adapted to delicately picking objects of similar shapes and sizes, but it might not be the best candidate for industrial applications, where versatility, compactness, robustness and simplicity are the main criteria.

The hand proposed by Hasegawa et al. (2017) consists of two underactuated fingers driven by a tendon. These fingers can adapt to the shape of the object to be picked. An extensible suction cup is embedded under the two fingers, sufficiently close to allow for combined grasps of larger objects. This suction cup is mounted on a revolute joint that allows to change its angle of approach when picking objects. This robotic hand seems versatile and particularly apt at grasping objects in tight spaces. In industrial applications, robustness considerations may require improvements and simplifications to its tendon drive and its suction cup swivel mechanism.

The gripper devised by Kang et al. (2019) includes two fingers, each with three phalanxes driven by two motors. The fingers are shape adaptive, as they tend to naturally wrap around the objects they encounter. Yet, they retain the capability of controlling the orientation of their distal phalanxes, which allows for parallel grasps. An extensible suction cup is embedded in the palm of the hand and driven by a pneumatic cylinder. This hand allows for combined grasps and can grasp objects of varied shapes and sizes. On the other hand, it is complex, involving four electric motors and one pneumatic cylinder. It will also have difficulty picking objects in tight spaces, because the fingers occupy a considerable volume on the sides of the palm as the suction cup is extended. This could prevent the gripper from accessing the objects close to the walls of a box, for instance.

### 1.3 Problem definition

In this article, we set out to design a gripper capable of grasping the widest variety of objects possible in industrial applications. Learning from previous grippers reviewed in Section 1.2, we list the postulates that guide us in the design process. The first postulate requires that the gripper be a combination of finger and suction cup. Second, to achieve a *combined grasp* or to transition between vacuum and finger grasps on the fly, the suction cup should be positioned in between the fingers. Third, the gripper is to be as narrow as possible, so that it can enter small and cluttered spaces. Fourth, in order to maximize the net payload of the robot, the gripper is to be as lightweight as possible. Fifth, again to reduce weight but also cost, only one actuator is permitted to drive the gripper mechanism. This objective should also help towards a mechanism simpler than most of those presented in the previous section. Sixth, for the sake of control simplicity, the tow distal phalanxes are to remain parallel at all times, a common trajectory used in many industrial grippers. Seventh, the design of the gripper must include the vacuum generator for the suction cup. The objective is to be able to integrate the gripper using only electricity without the need for an external vacuum generator that often requires pneumatic equipment. This gripper is intended to be the end effector of a collaborative robot such as the UR5 (Universal-Robots, 2023). This robot has an end-effector diameter of 75 mm and a payload of 5 kg. The class of objects to grasp is those having a mass smaller than 3 kg with one side smaller than 50 mm or a face susceptible to suction cups. Although these postulates do not specify what type of transmission nor general design is to be used, they are guidelines to follow while designing the gripper to ensure that the finished prototype has advantages and functionalities over some prototypes discussed in the previous section. In the next section of this article we discuss the transmission chosen for this particular gripper. This design process includes a conceptual approach leading to the proposed architecture, where the motions of the different parts of the gripper are described and how they are connected to the actuator. Then, design constraints are established to ensure functionality and manufacturability of the gripper. These constraints are presented as equations that can be used in a subsequent optimization phase. In this phase, performance parameters are defined and transmission parameters are adjusted to find the best configuration. Finally, the implementation of this design is presented and summarily evaluated with trials on a test set of 16 objects.

## 2 Transmission design

### 2.1 Proposed concept

**FIGURE 2:**
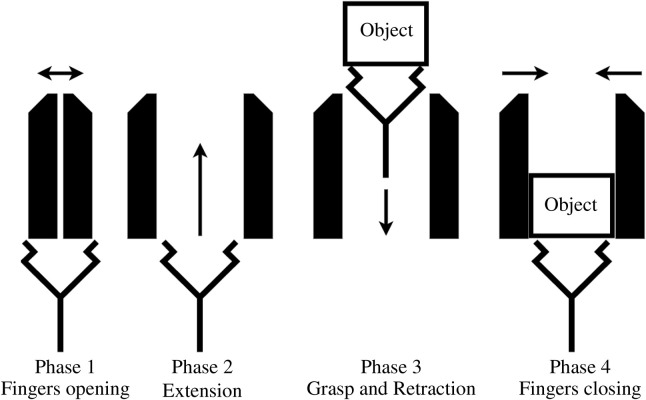
Fingers and suction cup motion.

In order to design from the objective and postulates established in Section 1.3, the desired motion of the fingers and suction cup is to be determined. If the suction cup is located in between the fingers, then only two approaches are possible to avoid any interference between these two components. The first is to have fingers that retract below the suction cup working surface (i.e., the palm of the hand). The second consists in using an extensible and retractable suction cup that reaches beyond the finger tips. In previous research (Courchesne and Cardou, 2021), the suction cup was fixed and a mechanism allowed the fingers to retract behind the palm so that the suction cup could reach the objects. This configuration was interesting but had a flaw: the mechanism that allows the finger to retract is bulky, occupying much space around the robot arm. To remedy this problem, the first approach is followed in this research, so that the suction cup is placed on a slider that allows it to reach beyond the fingers. As shown in Figure 2, the opening motion starts when the fingers are completely closed and follow a pure translation along a line perpendicular to the plane of symmetry of the hand. This motion stops when the hand is sufficiently open for the suction cup to pass through. The suction cup then moves out of the palm in a rectilinear motion as shown in Phase 2 of Figure 2. The closing motion is the exact reverse with the suction cup moving first (Phase 3 of Figure 2) and the fingers afterwards (Phase 4 of Figure 2). This opening and closing sequence is kept simple to facilitate the design and integration of the transmission using only one motor.

With two main parts moving, i.e., the fingers and the suction cup, a transmission that allows these parts to move in sequence is needed, while still using only one motor. Many solutions could be used to distribute the rotation of the motor to the moving parts, such as clutches, a solenoid plunger, a gear selector. But these solutions seem bulky, complex or difficult to integrate in a compact and slim design. In this study a differential planetary gear set is used. The planetary gear set offers many options for design with its three main parts all sharing the same rotation axis. In general, the planetary gear set is used as a reducer but it can also be used as a differential if properly integrated. It is composed of three main parts: the Sun gear, the planet carrier and the ring gear. Planetary gears are commonly used with one of these being fixed while the remaining two act as input and output, respectively. But with the carrier serving as the input and both the Sun and ring gear being allowed to rotate freely, then a differential behavior is obtained with the Sun and ring gears being the two outputs. An everyday application of a classic differential is seen in a car with a non-auto locking differential with one wheel one ice and the other on asphalt. The wheel with the lesser resistance—in this case, the wheel on ice—spins while the other remains blocked. The Sun and ring gears would then drive the two wheels and the carrier would be driven by the engine. In order to use this principle in the proposed gripper, we need a mechanism that locks and unlocks the Sun and ring gears to channel the motion appropriately to the suction cup or to the fingers. For this locking and unlocking mechanism it is planned to use end stops on the finger and suction cup axes. This is explained further in Section 3.1.

**FIGURE 3:**
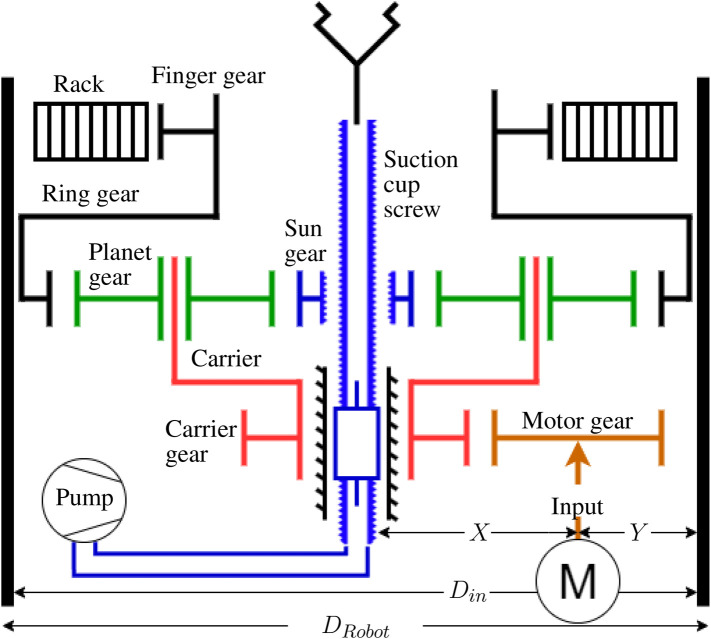
Transmission architecture.

Figure 3 is a schematic view of the transmission used in this gripper. The suction cup is mounted on a hollowed fast travel screw, also known as a multiple-start thread. The lower end of the screw is connected to a vacuum pump located inside the frame of the gripper. The screw is driven by a nut embedded in the Sun gear and its rotation is locked by parts in the lower section of the gripper. Hence, it can only move as a prismatic joint when the Sun gear is rotated. Because of this linear motion of the screw, the central axis of the gripper needs to be clear of any parts. This is why the motor is located at the side of the carrier, offset from centre. The fingers are mounted on symmetric racks that are driven by the finger gear, which is rigidly attached to the ring gear of the planetary gear set. In order to follow the motion prescribed, in the phase one of Figure 2, the fingers need to move first. Using end stops, the suction cup is blocked inside the palm, when the fingers are not fully open, prompting the planetary gear set to channel the motion to the fingers and not to the suction cup. Employing end stops again for the phase two of the motion, the fingers are blocked at the end of their travel while the suction cup is released, the Sun gear becoming free to rotate, allowing the suction cup to extend. Phases three and four use the same principles with the motor spinning in reverse.

The transmission architecture presented in Figure 3 does not provide ratios between the different gears. Some configuration could not perform well or even be manufacturable, and therefore more detailed design constraints are needed to obtain a functional gripper.

### 2.2 Design constraints

The architecture presented does not specify the number of teeth and diameter of each gear. Many arrangements of gears can produce a functional mechanism, many others could produce a non-functional or non-manufacturable mechanism. Design constraints are formulated to retain only functional mechanisms in the optimization process. These constraints are not related to the performance of the gripper. Their purpose is to limit the optimisation to combinations of teeth numbers that correspond to functional transmissions. The first constraint is the maximum outside diameter of the transmission; the second set of constraints guarantee a functional planetary gear set; the third constraint limits the size of the screw used in the shaft of the suction cup; the fourth constraint sets the maximum size of the motor and finally the fifth constraint limits the size of the rack and pinion used to move the fingers.

**FIGURE 4:**
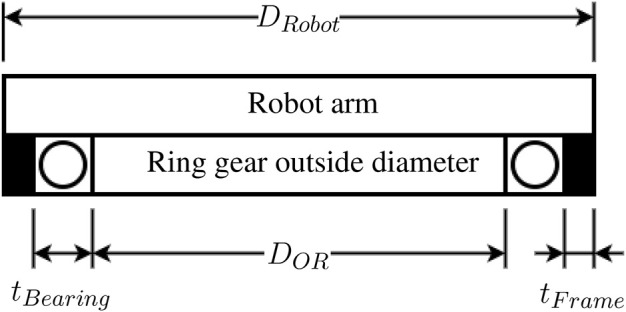
Ring gear packaging.

**FIGURE 5:**
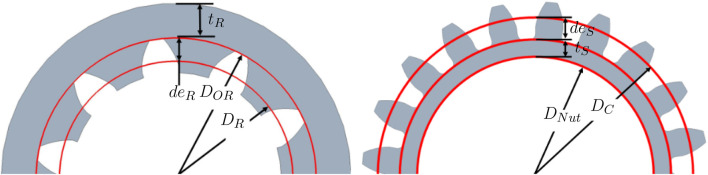
Ring gear and sun gear dimensions.

The first parameter considered in the dimensioning of the gears and general dimensions of the gripper is its maximum outside diameter. One of the design criteria is that the gripper should be narrow, allowing it to access tight spaces. The diameter of the gripper should thus be no larger than that of the robot on which it is mounted. In this case, the UR-5 robot is used as a reference. It has a diameter of 75 mm at its end effector; The maximum diameter of the gripper *D*_*Robot*_ is thus set to 75 mm. From this dimension, the maximum outside diameter of the ring gear *D*_*OR*_ is deduced while allowing space for the gripper housing thickness *t*_*Frame*_ and bearing thickness *t*_*Bearing*_. These dimensions are defined in Figure 4. Once the maximum outside diameter of the ring gear is established, it is converted into a pitch diameter and thence into a number of teeth which is more useful in calculations. The thickness after the teeth *t*_*R*_ and the teeth dedendum *de*_*R*_ of the gear are needed obtain the pitch diameter as shown in Figure 5. The thickness *t*_*R*_ is set to 2.75 mm for manufacturing and structural purposes. The dedendum is obtained from Eq. 2 and the gear module *m* one of the two discrete values 0.5 mm/tooth or 1 mm/tooth. The choice is restricted to these values because gears with these modules are largely available, saving us from custom gear fabrication.

$$D_{OR}=D_{\mathrm{Robot}}-2\left(t_{\mathrm{Frame}}+t_{\mathrm{Bearing}}\right)$$ (1)

$$de_{R}=1.25m$$ (2)

$$D_{R}=D_{OR}-2\left(t_{R}+de_{R}\right)$$ (3)

$$N_{R}=D_{OR}/m$$ (4)

The second constraints affect precisely the number of teeth of the planet and Sun gears. With the number of teeth of the ring gear being given by Eq. 4, the numbers of teeth of the Sun and planet gears can be computed. In order to get a functional planetary gear set, some constraints need to be satisfied. The first condition in Eq. 5 acts on the distance between the gear centres. If it is not satisfied, then the planet or Sun gears could be too large or too small to assemble and have a proper teeth mesh. The second condition expressed in Eq. 6 pertains to the symmetric placement of the planet gear. If this condition is not satisfied, then the planets cannot be arranged evenly around the Sun. The third condition in Eq. 7 prevents the interference between adjacent planet gears, where *P* is the number of planets. If this condition is not fulfilled, then the planet gears could interfere with one another. The fourth set of conditions limits the tooth count for the Sun and planet gears.

In Eq. 8, the minimum teeth count on every gear of the planetary is set to 12 and 28 for modules of 1.0 mm/tooth and 0.5 mm/tooth respectively. These values are some of the lowest found for a module of 0.5 or 1 in gear retailer catalogs. The maximum number of teeth in Eq. 9 is equal to the number of teeth of the ring gear. If custom gear fabrication is not a problem, then the modules and minimum teeth count can be adjusted accordingly. These four conditions are used in Section 2.3 to optimize the transmission while ensuring that the planetary gear set is functional.

$$2N_{P}+N_{S}=N_{R}$$ (5)

$$\frac{N_{S}+N_{R}}{P}\in N$$ (6)

$$N_{P}+2<\left(N_{S}+N_{P}\right)\sin\left(\pi /P\right)$$ (7)

$$N_{S},N_{P}⩾\left\{\begin{array}{cc} 12,\; & \text{if}\;m=1.0\\ 28,\; & \text{if}\;m=0.5 \end{array}\right.$$ (8)

$$N_{S},\;N_{P}⩽N_{R}$$ (9)

The third constraint limits the size of the suction cup screw. As shown in Figure 3, the suction cup screw is driven by a nut located inside the Sun gear. Therefore, the diameters of the screw and screw nut cannot be too large or else they will interfere with the Sun gear teeth. To prevent this interference, a maximum diameter for the screw nut is established using Eq. 10, where *D*_*S*_ is the Sun gear pitch diameter, *de*_*S*_ the Sun gear dedendum and *t*_*S*_, the minimal thickness at the base of the Sun gear teeth. Nuts for fast travel screws are available in various sizes and shapes, such as flanged, external threaded and rounded. In this case an externally threaded nut is used. Hence, if the maximum diameter obtained in Eq. 10 is larger than the major diameter of the threaded nut, the nut and its corresponding screw can be selected.

$$D_{\mathrm{Nut}}=D_{S}-2\left(de_{S}-t_{s}\right)$$ (10)

The fourth constraint concerns the size and placement of the motor and is related to the overall layout of the transmission. With the motor placed at the side of the carrier as shown in Figure 3, its position is influenced by the number of teeth of the carrier gear and motor gear. The minimum pitch diameter of the carrier gear is defined by Eq. 11 where *D*_*Screw*_ is the outside diameter of the suction cup screw, clearance *F* is the space between the suction cup screw and the centre hole of the carrier gear, and base *B* is the material thickness under the tooth profile of the carrier gear. With the carrier gear pitch diameter, the pitch diameter of the motor gear can be solved while maximizing the motor diameter. From Figure 3, the motor diameter is maximized when *X* and *Y* are equal. With this constraint we ensure that the largest motor possible can be used, resulting in the greatest torque for the finger grasp. Using the pitch diameter of the carrier gear, the screw diameter and interior diameter of the frame, the pitch diameter of the motor gear is obtained through Eq. 12. This combination of carrier gear and motor gear ensures that the motor can have the largest diameter possible. In the optimization phase, the pitch diameter of the carrier can be increased to obtain a different gear ratio between the motor gear and carrier gear while keeping the largest motor possible using Eq. 12.

$$D_{\mathrm{Cmin}}=D_{\mathrm{Screw}}+Freeplay+Base+1.25m$$ (11)

$$D_{M}=\frac{D_{in}+D_{\mathrm{Screw}}}{2}-D_{C}$$ (12)

The fifth constraint expressed in Eq. 13 applies to the size of the finger gear. It is similar to the constraint of the carrier gear pitch diameter explained in Eq. 11. The screw of the suction cup must be able to pass through the finger gear as shown in Figure 3. The pitch diameter obtained is thus the minimum allowed by the screw and can be increased if needed. Furthermore, the minimal number of teeth of the finger gear is rounded up to the nearest even integer to ensure that both finger racks can be identical. This also avoids any positions of the fingers that would be caused by an odd number of teeth on the finger gear. This constraint can be removed if the design and fabrication of asymmetric fingers is not a problem.

$$N_{F}\geq N_{\mathrm{Cmin}},\;N_{F}/2\in N$$ (13)

These constraints can now be used in an optimization algorithm. They limit the number of solution that the algorithm can produce and ensure that every solution found is functional.

### 2.3 Optimization

In the previous Sections 2.1, 2.2, the architecture of the transmission was established and constraints ensuring its proper functioning were expressed mathematically. Using the design constraints, an optimization algorithm is devised to compute the performance of every possible solution. The algorithm returns a matrix where every line is a functional solution and where the columns correspond to specifications of the solutions (e.g., dimension, speed, force, etc.). Every tooth count for the planetary gear set is recorded in this matrix, providing the designer with all the data needed to make the gripper. This matrix can then be presented as a graph, table or chart using the desired parameters. The resulting matrix is also coupled to CAD software. The CAD assembly is then updated if the matrix changes or if a different solution is chosen. This coupling allows automated easy and quick visualisation of a solution. The goal of this optimization is to allow the designer to choose the solution that best meets his or her requirements from a chart, table or graph. The performance criterion can be decided by the designer. If the opening speed has to be maximized, then the chosen solution would be the fastest of the graph.

**FIGURE 6:**
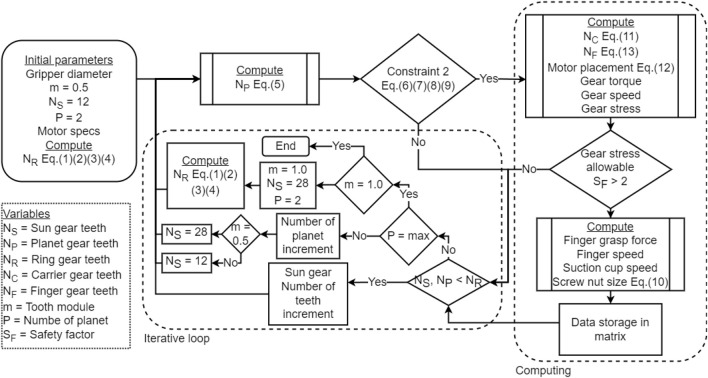
Optimization algorithm.

The algorithm uses an iterative logic that sweeps through the possible solutions while only retaining the functional ones. With each iteration, a variable is incremented or changed. This algorithm is represented in Figure 6. Using the initial parameters *m*, *N*_*S*_ and *P* described in Section 2.2, the number of teeth of the ring and planet gears, *N*_*R*_ and *N*_*P*_ are computed. If the second set of constraints composed of Eqs 6–9 is satisfied, then the algorithm enters the dashed lines. These operations compute additional parameters for the planetary gear set, some performance metrics and stores these data in a matrix. Once a solution has been stored in the matrix or rejected, the iterative process starts. The first variable to be incremented is the Sun gear tooth count *N*_*S*_, then the planet gears count and finally the teeth module. Once the number of planets and module have been incremented, every combination allowed by the design constraints has been evaluated by the algorithm and the loop ends. The data obtained in the matrix can then be organised by the designer to reflect the needs of a specific application or general desired performance.

**FIGURE 7:**
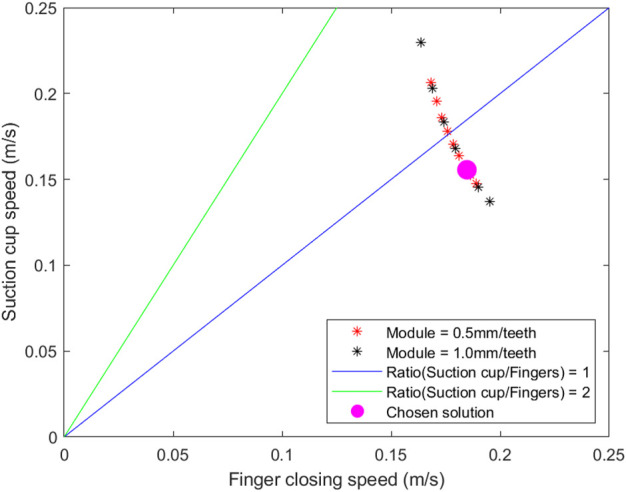
The finger and suction cup speeds of all the possible transmission systems.

In this research, the performance criterion chosen is the speed ratio between the suction cup and the finger, which is to be as close to one as possible. Thus the opening and closing speed of the fingers and that of the suction cup extension and retraction should be similar. Having a slow motion of the suction cup would be impractical for a combined grasp of objects, as it increases transition time between the suction and finger grasps. The graph in Figure 7 is traced using all the possible solutions with the speeds of the suction cup and fingers as horizontal and vertical axes. The configuration we chose to build in this case is a proof of concept and not the only viable solution. It is represented as a pink dot in Figure 7. Some solutions had a ratio closer to one but this configuration was chosen because it is easily manufactured with as many standard parts as possible.

Once the solution is chosen, the number of teeth of each gear is known but it is still not ready for manufacturing. Bearings, fasteners, motor and other components have to be selected. The motor specifications are integrated with the initial parameters in the algorithm. The designer can then adjust the specifications of the motor until the desired performance is achieved. In this study the chosen solution has a finger speed of 0.18 m/s and a theoretical finger grasping force of 73 N. In the next section, we discuss the implementation of this solution into a functional prototype.

## 3 Implementation

### 3.1 Prototype embodiment

**FIGURE 8:**
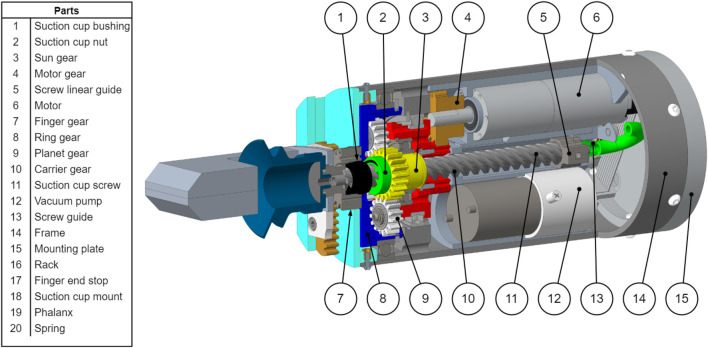
Design partial section view.

With the design constraints an optimization completed, all the essential parameters to design a functional gripper are known. A concept of the gripper proposed in this study is presented in Figure 8. It has a cylindrical shape with a diameter of 75 mm as prescribed in Section 2.2. The design presents a hollow Sun gear (3) that allows the suction cup screw to slide along its center axis. The motor (5) is placed off centre and uses the aluminum frame (13) as a heat sink. The chosen motor is rated for continuous operation with the desired finger grasp force of 73 N. A vacuum pump is placed off centre and connected to a solenoid valve that is not visible in Figure 8. The valve is then connected by a flexible tube to the hollow suction cup screw (10).

**FIGURE 9:**
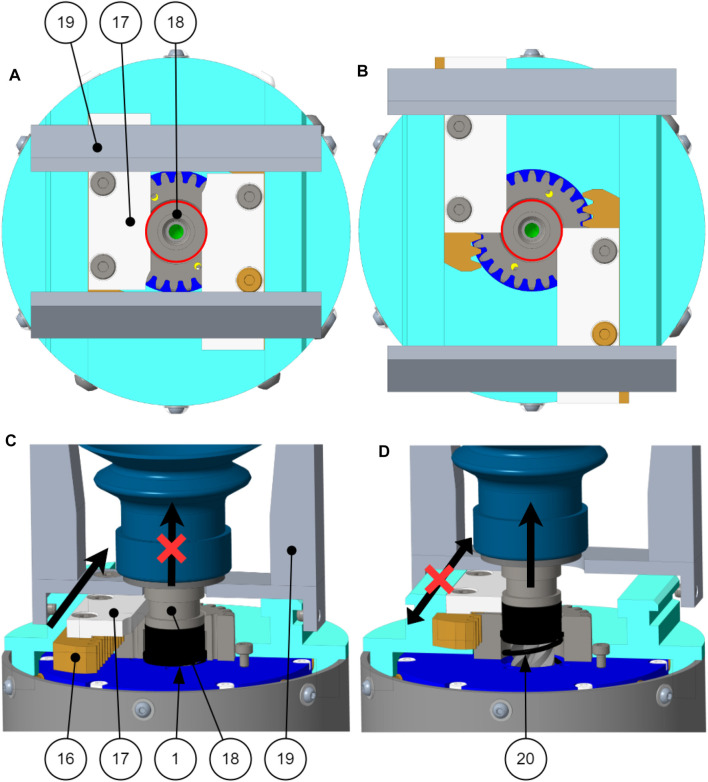
End stops mechanism **(A)** Phase 1 and 4 in top view. **(B)** Phase 2 and 3 in top view. **(C)** Phase 1 and 4. **(D)** Phase 2 and 3.

The sequential motion for the fingers and suction cup is presented in Figure 2. As presented in Figure 3, the fingers are driven by the ring gear, the suction cup is driven by the Sun gear and the carrier is the motion input. The following references uses the part number shown in Figures 8, 9. End stops are used to channel the motion of the motor in sequence to the fingers and the suction cup. Firstly, when the fingers are opening and closing, the suction cup has to remain in the retracted position. Secondly, when the suction cup is extending and retracting, the fingers have to remain in the fully open position. The end stops are used to lock the Sun gear (3) when the fingers are moving and the ring gear (8) when the suction cup is moving.

In phase 1 of the motion, the fingers are opening. The phalanxes (19) are mounted on gear racks (16) and acetal end stops (17). The end stop (17) slides into a groove located on the suction cup mount (18). When the finger is not completely open as shown in Figures 9A, C, the finger end stops (17) prevent the suction cup from moving out of the palm.

In phase 2 of the motion, the suction cup extends from the palm of the gripper. When the fingers are fully open as show in Figures 9B, D, the fingers end stops (17) are no longer into the groove of the suction cup mount (18), the suction cup can then extend from the palm. As the suction cup leaves its retracted position, the suction cup bushing (1) is pushed out of the palm by a compression spring (20).

In phase 3 of the motion, the suction cup retracts into the palm of the gripper. The bushing (1) prevents the fingers from closing while the suction cup is retracting because the bushing blocks the fingers end stops (17) from sliding.

**FIGURE 10:**
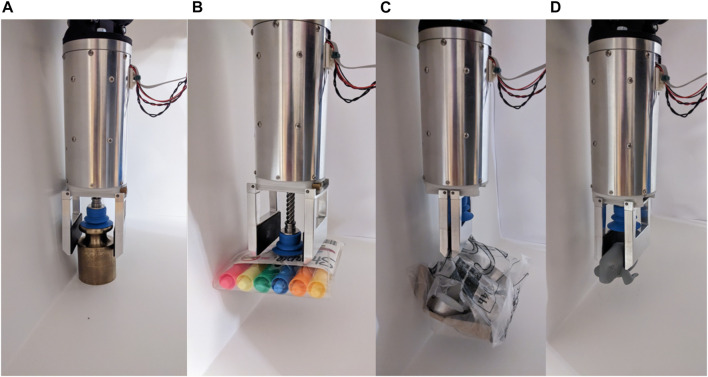
Grasps of objects **(A)** Brass weight using combine grasp. **(B)** Highlighter bag using vacuum grasp. **(C)** Bagged item using parallel grasp. **(D)** Rubber toy using parallel grasp.

In phase 4 of the motion, the fingers are closing. As long as the bushing is not pushed back into the palm, the fingers cannot close. The only way to push the bushing back into the palm is by retracting the suction cup enough to compress the bushing (1) and spring (20). When the suction cup is fully retracted, the groove of the suction cup mount (18) is aligned with the fingers end stops (17) and the fingers can close Figure 10.

### 3.2 Prototype evaluation

A prototype of the proposed concept is fabricated according to the parameters of the chosen solution indicated on Figure 7. This solution was obtained by applying all the design constraints outlined in Section 2.2 and the optimization method presented in Section 2.3. It is therefore functional and has known gear characteristics, forces and speeds, which are shown in Table 1. This solution was chosen to facilitate the manufacturing of the prototype. Other solutions of Figure 7 needed gear tooth numbers that were not available on the market at the time of the build. All gears of the chosen solution could be purchased off the shelf except for the ring gear.

**TABLE 1:** Prototype specifications.

Parameter	Value	Unit
Number of planet	5	-
Number of ring gear teeth	50	-
Number of sun gear teeth	20	-
Number of planet gear teeth	15	-
Number of carrier gear teeth	20	-
Number of motor gear teeth	21	-
Number of finger gear teeth	24	-
Finger opening width	0.05	m
Finger and suction cup speed ratio	0.84	-
Gear maximum stress	66.4	MPa
Motor maximum diameter	0.031	m
Screw maximum diameter	0.0138	m

**FIGURE 11:**
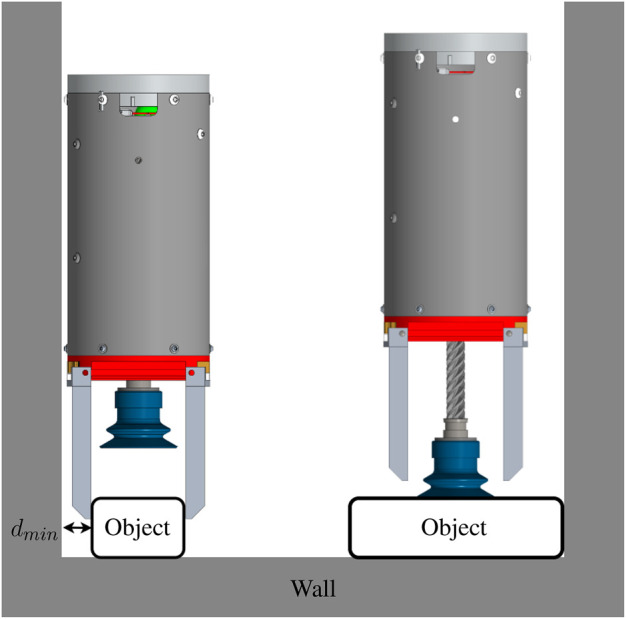
Grasping objects in bin or box with objects close to walls.

In a context of production of this gripper, purchasing only available standard components can be an important design criterion. Some minimal modifications were made to the gears of the prototype, e.g., modifying the diameter of the bore for a specific bearing at the centre of the gear. The overall system of transmission of the torque from the motor to the finger gear has only four custom parts that needed to be machined from scratch: the motor housing, the planet carrier, the ring gear and the gripper frame. The motor housing, carrier and gripper frame were machined using simple three-axis lathe and milling. The ring gear was machined using an EDM wire cutter Figure 11.

The built prototype performances are evaluated in two parts. The first part is a quantitative evaluation of the grasping strength and of the speeds of the suction cup and fingers. The measured results are presented in Table 2. The first observation made is that the measured grasping strength is significantly lower than that which was specified. We identified two reasons that could explain this discrepancy. Firstly, the theoretical grasping force was calculated without considering gear efficiency. Secondly, a manufacturing error on the ring gear was noticed after assembly. The root diameter is slightly smaller than what it is supposed to be. This smaller root diameter of the ring gear compresses the planet gear nylon teeth, which dramatically increases friction between these gears and also the torque required to turn them. In future work, the ring gear is to be replaced with a new one of proper dimensions.

**TABLE 2:** Prototype speeds and strength.

Parameter	Predicted	Measured	Unit
Finger grasping force	74	25	N
Suction cup maximum speed	0.156	0.093	m/s
Finger maximum speed	0.185	0.050	m/s

The measured speed is also lower than the predicted maximum speed due to the control of the gripper. The speed of the gripper was reduced by adjusting the controller to have slower and steadier grasp on objects.

The third objective of this research is that the gripper should be as narrow as possible to enter small and cluttered spaces. To assess the performance of the gripper with respect to this objective, objects are positioned along a wall, as shown in Figure 11. This configuration simulates an object being placed inside a box. Table 3 presents the results of this test and Figure 10 depicts a few of the successful grasp of these objects. The width specified is the smallest width of that object. The distance *d*_min_ is the minimum distance from the wall at which the gripper can grasp the object. A small distance is a sign of good performance of the prototype. The minimum distance observed in this test is 15 mm, this distance corresponds to the thickness of the phalanx. An arbitrarily thin phalanx would make it possible to grasp objects at an equally small distance from the wall. The objects of Table 3 that the gripper failed to pick up have common features. Their minimum widths are larger than the opening width of the fingers, while their surfaces are porous, preventing a grasp using the suction cup. It is probable that a more powerful vacuum generated by a pneumatic system on the ground would have allowed successful grasps on almost all the objects in the test sample.

**TABLE 3:** Results of grasping trials with 16 different objects.

Object	Object width (mm)	Weight (g)	Grasp type	Distance between object and wall (mm)
Plush cylinder	130	75	Failed	-
Foam block	110	20	Failed	-
Cardboard	92	40	Failed	-
Bagged item	175	150	Parallel	15
Spray bottle	35	60	Parallel	0
Highlighter bag	125	80	Vacuum	0
Highlighter box	140	180	Vacuum	0
CD box	123	36	Vacuum	0
Tenis ball	67	57	Failed	-
Rubber toy	50	25	Parallel	15
Post it stack	51	20	Parallel	15
Credit card	54	5	Vacuum	0
PCB	65	80	Parallel	15
Rubber sheet	80	15	Vacuum	0
Brass weight	51	1,000	Vacuum, parallel and combined	15
Steel plate	210	3,000	Vacuum	0

This prototype can be compared to some widely used commercial grippers such as the *Hand-E* of Robotiq. (2020b) which uses linear finger motion and the vacuum gripper *Epick* of Robotiq. (2020c), which also uses an integrated vacuum generator. Table 4 presents the main specifications of these grippers. As can be appreciated form this comparison, a relatively small amount of additional weight allows to combine finger and vacuum grasps in a single gripper. The strength of our prototype is somewhat inferior to those of the Robotiq. (2020b) and the *Epick* but some mechanical adjustments and minor changes would narrow or erase this gap. Finally, the operation of the prototype can be appreciated in the accompanying video. Notice that the trials shown in the video are not those that are reported in Table 3.

**TABLE 4:** Prototype specifications compared to market products.

Parameter	Hand-E	Epick	Prototype	Unit
Energy source	Electricity	Electricity	Electricity	-
Gripper mass	1,000	710	1,300	g
Vacuum level	N/A	−80	−60	kPa
Stroke	50	N/A	50	mm
Grip force	20 to 185	N/A	25	N
Closing speed	20 to 150	N/A	50	mm/s

## 4 Conclusion

In this research the first objective is to integrate finger grasp and vacuum grasp into a single gripper. The proposed architecture and its transmission based on a planetary gear set allow to switch between finger, vacuum and combined grasps on the fly, which is the second objective. The third objective is that the gripper be as narrow as possible; With a diameter of 75 mm, this prototype is as narrow as many robotic arm end effectors. The fourth objective is to be as lightweight as possible; With a mass of 1.3 kg, it is on par with similar grippers. The fifth objective is to use only one actuator to drive both the fingers and the suction cup. This is achieved through the proposed planetary gear set, which acts as a differential, as detailed in Section 2. The sixth objective is to have the distal phalanxes remain parallel at all times during the closing and opening of the fingers. This is achieved through a rack and pinion mechanism driving both fingers. Finally, a vacuum generator is integrated in the frame of the gripper for the suction cup, this results in a gripper using electricity as its only source of energy.

**FIGURE 12:**
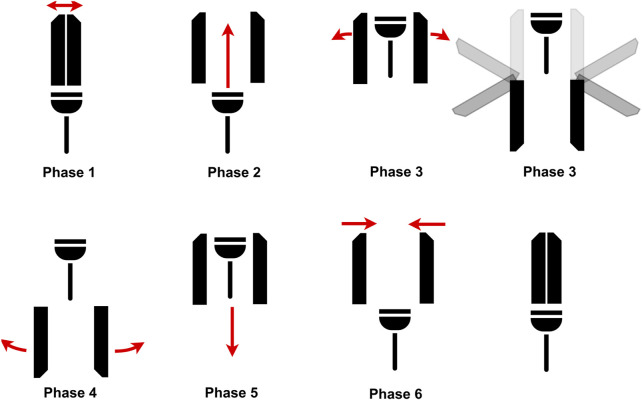
Different desired phases.

The produced gripper is versatile and can grasp a wide variety of objects, as is illustrated in Section 3 by grasping a sample of 16 objects. 12 of these objects were grasped successfully. The remaining four could probably be grasped with a stronger vacuum system. There are some shortcomings to this design, however, one them being that the suction cup diameter is close to the opening width of the fingers. A smaller suction cup would allow for a larger clearance between the fingers and the suction cup. This would make it easier to vacuum grasp objects without interfering with the fingers. An even more effective method of minimizing such interferences between the fingers and the grasped objects would be to implement the finger retraction mechanism, which is the topic of ongoing research in our laboratory. The purpose of this mechanism would be to fold the fingers back on the gripper frame when they reach their fully open positions. The new opening and closing sequence of the hand, shown in Figure 12, would thus be performed in six phases instead of the four presented in Figure 2. This mechanism could not be tested for want of time, but promises to eliminate the chances of an unwanted collision between fingers and object.

The most challenging constraint encountered in this research is the maximum diameter of 75 mm, which includes the finger width. This constraint allowed a finger stroke of 0–50 mm. This stroke can be too small to grasp larger objects without using the suction cup. However, the design of the transmission allows to change the finger phalanxes without changing the rack used to drive them. Hence the stroke could be offset to accommodate special cases. For example, one could devise fingers for a stroke of 50–100 mm that allows to grasp larger objects.

In summary, this research led us to a gripper design that integrates the two grasping strategies that are most popular in industry: finger grasps and vacuum grasps. Combining these two technologies appears to be an efficient approach to increasing the versatility of current grippers while relying on tried and tested methods. While this is not the first time researchers attempt such a combination, we believe that it needs to be further explored while keeping in mind the successes and failures that took place in real-life applications. In particular, it is the belief of the authors that one should not err too far on the side of complexity, but rather tinker with the delicate balance between qualities appreciated in industry such as versatility, reliability, compactness and cost effectiveness. Inventiveness and creativity can be used to make gains on some of these qualities without sacrificing the others.

## Data Availability

The original contributions presented in the study are included in the article/supplementary material, further inquiries can be directed to the corresponding author.
